# Automatic identifying and counting blood cells in smear images by using single shot detector and Taguchi method

**DOI:** 10.1186/s12859-022-05074-2

**Published:** 2022-12-08

**Authors:** Yao-Mei Chen, Jinn-Tsong Tsai, Wen-Hsien Ho

**Affiliations:** 1 School of Nursing, Kaohsiung Medical University, Kaohsiung, 807 Taiwan; 2 Superintendent Office, Kaohsiung Medical University Hospital, Kaohsiung, 807 Taiwan; 3 Department of Computer Science and Artificial Intelligence, National Pingtung University, Pingtung, 900 Taiwan; 4 Department of Healthcare Administration and Medical Informatics, Kaohsiung Medical University, Kaohsiung, 807 Taiwan; 5 Department of Medical Research, Kaohsiung Medical University Hospital, Kaohsiung, 807 Taiwan

**Keywords:** Platelets, Red blood cells, White blood cells, Single shot detector, Resnet50 model, Taguchi experimental method

## Abstract

**Background:**

Researchers have tried to identify and count different blood cells in microscopic smear images by using deep learning methods of artificial intelligence to solve the highly time-consuming problem.

**Results:**

The three types of blood cells are platelets, red blood cells, and white blood cells. This study used the Resnet50 network as a backbone network of the single shot detector (SSD) for automatically identifying and counting different blood cells and, meanwhile, proposed a systematic method to find a better combination of algorithm hyperparameters of the Resnet50 network for promoting accuracy for identifying and counting blood cells. The Resnet50 backbone network of the SSD with its optimized algorithm hyperparameters, which is called the Resnet50-SSD model, was developed to enhance the feature extraction ability for identifying and counting blood cells. Furthermore, the algorithm hyperparameters of Resnet50 backbone networks of the SSD were optimized by the Taguchi experimental method for promoting detection accuracy of the Resnet50-SSD model. The experimental result shows that the detection accuracy of the Resnet50-SSD model with 512 × 512 × 3 input images was better than that of the Resnet50-SSD model with 300 × 300 × 3 input images on the test set of blood cells images. Additionally, the detection accuracy of the Resnet50-SSD model using the combination of algorithm hyperparameters got by the Taguchi method was better than that of the Resnet50-SSD model using the combination of algorithm hyperparameters given by the Matlab example.

**Conclusion:**

In blood cell images acquired from the BCCD dataset, the proposed Resnet50-SSD model had higher accuracy in identifying and counting blood cells, especially white blood cells and red blood cells.

## Background

A complete blood count can help diagnose certain cancers, infections, anemia, and many other diseases, and monitor drug side effects [1]. Medical laboratories have large amounts of tissue samples and large amounts of blood that need to be analyzed in the shortest possible time. The ability to quantify specific cell populations is important for accurate diagnosis. Counting blood cells is a time-consuming and tedious task that must be counted manually using hemocytometers, laboratory equipment, and chemical compounds. Medical staffs often work overtime to analyze all samples, resulting in high staff fatigue, which can lead to errors and reduce work efficiency [2]. These mistakes can have serious or even fatal consequences for treating patients. Automatic detection and counting of blood cells in images is a complex task where the resolution of medical images can be very high and the target cells become very dense. In addition, the number of blood cells in the medical image is high, and the blood cells often overlap, making it a problem to distinguish blood cells [3]. Therefore, artificial intelligence models for automatically detecting and counting blood cells are needed.

### Literature review

In recent years, the method of deep learning networks has become one of the primary methods for many computer vision applications in medical images, such as classification, segmentation, or object detection of medical images. Deep learning methods in medical image detection were effective and applied to different applications and studies in many medical image detection cases, including studies for automated blood cell counting [4].

Alam and Islam [5] proposed the YOLO (You Only Look Once) algorithm of the deep learning method to automatically identify and count three types of blood cells. The YOLO model has been trained using the changed blood smear images of BCCD dataset to automatically identify and count white blood cells (WBCs), red blood cells (RBCs), and platelets. The proposed YOLO model can accurately count some unlabeled cells in the dataset, and was also tested on different smear image datasets with satisfactory results. Acevedo et al. [6] proposed a classification method by using a trained convolutional neural network to distinguish between eight types of blood cells. The study used two convolutional neural network architectures, Inceptionv3 and VGG-16. First, convolutional neural networks were used as feature extractors, and these features were used to train support vector machine classifiers. Next, the convolutional neural network was fine-tuned to obtain two models for classifying eight types of blood cells. Wang et al. [7] applied two excellent object detection methods, YOLO and single shot detector (SSD), for leukocyte identification. Since the classification is based on automatic feature extraction of convolutional neural networks, segmentation is not required, but it is difficult to handle multi-target recognition.

Alzubaidi et al. [8] used deep learning models to classify different RBCs. The problem of lack of training data in the RBCs classification task was solved by using two techniques, data augmentation and transfer learning. The proposed model efficiently extracted features by using a multi-class support vector machine classifier. Loey et al. [9] proposed two classification models to classify leukemia in microscopic images. The two models using transfer learning had several advantages over traditional methods. Reena and Ameer [10] proposed a two-stage pipelining including semantic segmentation and transfer learning for classification. The study used a pre-trained network to segment leukocytes and used AlexNet to classify five types of leukocytes in peripheral blood from microscopic smear images.

Khan et al. [11] proposed the AlexNet multilayer convolutional neural network model for WBC type recognition. Parab and Mehendale [12] proposed a classification method that combined with image processing to help convolutional neural networks to classify RBCs. The classification method can extract the features of each segmented cell image and classify them into 9 different types. Vogado et al. [13] proposed a convolutional neural network named LeukNet, inspired by the VGG-16 convolutional networks, for diagnosing leukemia in blood slices. Data augmentation was applied to expand the training dataset. Chen et al. [14] proposed a Resnet101-9 ensemble model with a majority voting strategy for classifying acute lymphoblastic leukemia in microscopic images. Each trained Resnet-101 model combined a pre-trained Resnet-101 model and its algorithm hyperparameters to classify acute lymphoblastic leukemia. The Taguchi method found the best algorithm hyperparameters combination for the pre-trained Resnet-101 model. Drałus et al. [3] proposed the RetinaNet deep learning network to identify and count three types of blood cells in microscope smear images. The trained network can automatically identify and count platelets, WBCs, and RBCs. Furthermore, the trained model was tested on different smear images, and showed promising results. However, the accuracy of counting depended on choosing the proper confidence threshold for each cell class.

From the above literature review, most papers used deep learning methods to classify blood cells, and only a few papers used deep learning methods to detect and count RBCs, WBCs, and platelets simultaneously [3, 5]. Few studies discussed how the combination of algorithm hyperparameters affects the detection accuracy in a backbone network of deep learning methods [14, 15]. The image size and the object size and number are related to the accuracy of detection and are also worth discussing during object detection. Therefore, the motivations of this study are on the detection of multi-blood cell objects, on the accuracy of the image size and the object size and number, and on the impact of the combination of algorithm hyperparameters on the detection accuracy of the backbone network of deep learning methods.

### Objectives

This study had two goals. The first goal was to find the best combination of algorithm hyperparameters for the Resnet50 backbone network of the single shot detector (SSD), which is called the Resnet50-SSD model, for automatically identifying and counting different blood cells. The second goal was to understand the image size and the object size and number to improve detection accuracy. In the Resnet50-SSD model, detection quality depended on algorithm hyperparameters combination before the learning process started. In consecutive training, the Resnet50 backbone network of the SSD may require different algorithm hyperparameters combination to improve detection accuracy. This study used Taguchi experimental method, a systematic and robust method, to find the best algorithm hyperparameters combination for the Resnet50-SSD model for automatically identifying and counting different blood cells. In experimental results, the Resnet50-SSD model had higher detection accuracy compared with the previous models and had better accuracy in identifying and counting different blood cells.

### Problem description

The three main cells that make up blood are WBCs, RBCs, and platelets. The number of WBCs is 4,500 to 11,000 per microliter in the blood. Normal WBCs have a diameter of 12–15 μm. WBCs are the largest of the blood cells but also the fewest. WBCs are also called leukocytes, and they fight infections. Standard ranges for RBCs are 4.2 to 5.4 million per microliter for females and 4.7 to 6.1 million per microliter for males. Normal RBCs have a diameter of 6–8 μm. RBCs, also known as erythrocytes, carry oxygen to our body tissues, and the number of RBCs affects the amount of oxygen that tissues receive. Platelets are also known as thrombocytes, and they help with blood clot. The number of normal platelets ranges from 150,000 to 450,000 per microliter in the blood. A normal platelet has a diameter of 2–3 μm. A complete blood count is an important test for medical professionals to assess the health conditions [16, 17]. Because of the large number of blood cells, traditional manual blood cell counting systems using hemocytometers have a high error rate and are time-consuming, and the accuracy in most cases depends heavily on the skills of the clinical laboratory analyst [18, 19]. Therefore, an automated process to identify and count different blood cells from smear images would aid the entire counting process. To help medical laboratory personnel identify and count different blood cells, artificial intelligence models developed by deep learning methods could be a useful tool.

**Fig. 1:**
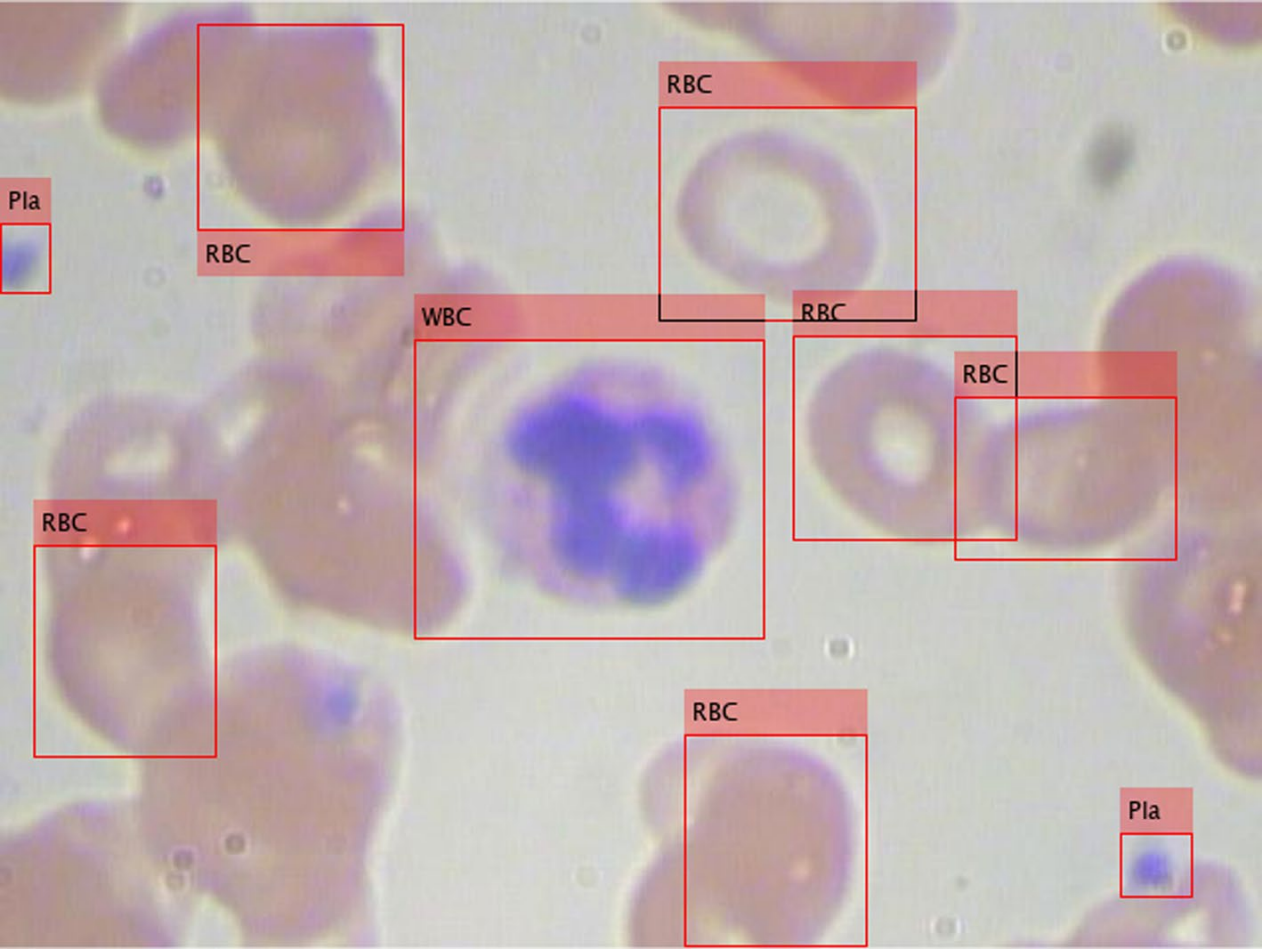
Representative smear images of blood cells

A publicly available dataset of annotated blood cell images, known as the blood cell counting and detection (BCCD) dataset, was employed. The BCCD dataset is a small-scale dataset for blood cells detection with 364 annotated smear images. The BCCD dataset has three classes of RBC, WBC, and platelet, and the width and height of the JPEG smear image are 640 and 480 pixels, respectively [20]. There are 4,888 labels across 3 classes, including 4,155 RBCs, 372 WBCs, and 361 platelets labels. Figure 1 shows representative smear images of blood cells. A platelet is only about 20% in the diameter of a RBC. A WBC is about twice the diameter of a RBC. The diameter ratio of platelets, RBCs, and WBCs is 0.2:1:2.

## Results

The proposed Resnet50-SSD model used the Resnet50 network as a backbone network of the SSD with its optimized algorithm hyperparameters to enhance the feature extraction ability for detecting and counting different blood cells in smear images. The Resnet50-SSD model was trained by using a training set of smear images of different blood cells from the BCCD dataset. Performance evaluation of the Resnet50-SSD model used a test set of smear images of different blood cells from the BCCD dataset. The experimental environment was the computer of Intel i7 CPU and Turbo-RTX2080Ti-11G GPU with Matlab R2021a developed by MathWorks and its toolboxes.

### Image data preparation

The experimental smear images used to test performance in detecting and counting different blood cells included the training and test sets. The study used 364 smear images of different blood cells and labeled different blood cells for object detection. Each experiment randomly selected 291 images (80% of all images) as the training set and 73 images (the remaining 20% images) as the test set for identifying and counting different blood cells. The ground truth labels for the training and test sets were created for evaluating the detection accuracy. To efficiently detect objects and fit into the Resnet50 backbone network of SSD, each image was processed as 512 × 512 × 3 image size, where 3 is the number of color channels.

**Fig. 2:**
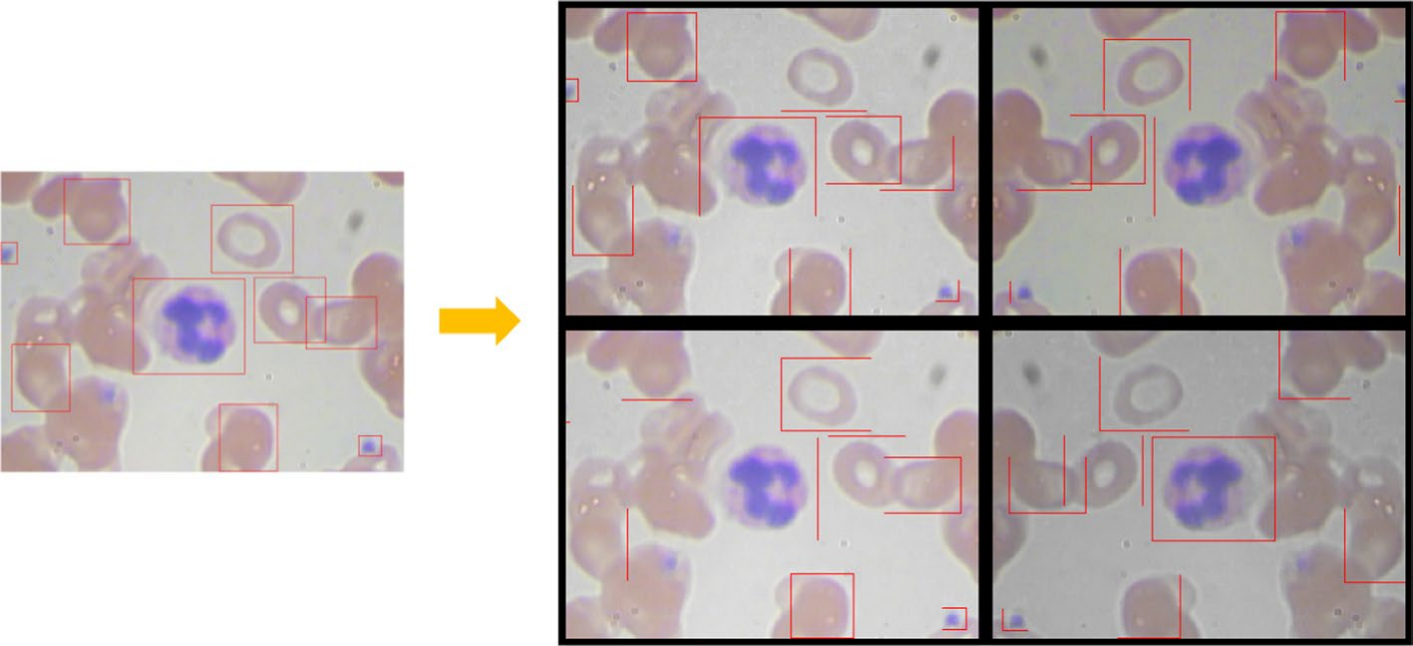
An example of a smear image of different blood cells of data augmentation

Data augmentation strategies included randomly scaling the image and associated box labels, randomly flipping the image and associated box labels horizontally, and using jitter image colors. Data augmentation was used to the training data, and was not adopted to the test data, which should represent the original data and be unmodified for unbiased evaluation. Figure 2 shows an example of a smear image of different blood cells of data augmentation.

### Taguchi experimental design for the algorithm hyperparameters combinations for the Resnet50 backbone network of the SSD

The three-level OA for the minimum number of experiments for the four factors is the three-level *L*_9_(3^4^). Table 1 shows the three-level *L*_9_(3^4^) OA, and Table 2 shows the factors and levels for the Resnet50 backbone network of the SSD. The three levels for the hyperparameter ‘Optimizer’ (factor A) were ‘adam (adaptive moment estimation)’, ‘sgdm (stochastic gradient descent with a momentum)’, and ‘adam’. The three levels for the hyperparameter ‘MiniBatchSize’ (factor B) were 14, 16, and 18, because of the GPU memory limit. The three levels for the hyperparameter ‘InitialLearnRate’ (factor C) were 10^−3^, 10^−4^, and 10^−5^. The three levels for the hyperparameter ‘LearnRateDropPeriod’ (factor D) were 30, 40, and 50. Instead of 81 (3^4^) experiments, the *L*_9_(3^4^) OA required only 9 experiments.

**Table 1:** *L*_9_(3^4^) OA

Experiment no	Factors			
	A	B	C	D
1	1	1	1	1
2	1	2	2	2
3	1	3	3	3
4	2	1	2	3
5	2	2	3	1
6	2	3	1	2
7	3	1	3	2
8	3	2	1	3
9	3	3	2	1

**Table 2:** Factors and levels for the Resnet50 backbone network of the SSD

Factor (algorithm hyperparameter)	Levels		
	1	2	3
A: Optimizer	adam	sgdm	adam
B: MiniBatchSize	14	16	18
C: InitialLearnRate	10^−3^	10^−4^	10^−5^
D: LearnRateDropPeriod	30	40	50

Table 3 shows the four algorithm hyperparameters combinations that combined the values in Tables 1 and 2. The algorithm hyperparameters combinations were used in the Resnet50 backbone network of the SSD for detecting and counting different blood cells in smear images.

**Table 3:** Four algorithm hyperparameters combinations for the Resnet50 backbone network of the SSD

Experiment no	Algorithm hyperparameters			
	Optimizer	MiniBatchSize	InitialLearnRate	LearnRateDropPeriod
1	adam	14	10^−3^	30
2	adam	16	10^−4^	40
3	adam	18	10^−5^	50
4	sgdm	14	10^−4^	50
5	sgdm	16	10^−5^	30
6	sgdm	18	10^−3^	40
7	adam	14	10^−5^	40
8	adam	16	10^−3^	50
9	adam	18	10^−4^	30

### Conducting detecting experiments and recording blood cells detection performances of the Resnet50-SSD model on smear images of different blood cells

The combinations of four algorithm hyperparameters in Table 3 were employed in independent experimental runs in the training and test sets for the Resnet50 backbone network of the SSD. In tests of performance in detecting smear images of different blood cells, Table 4 shows the mAP got in a single run, and the average mAP, standard deviation (SD), and *η* value got in three independent experimental runs.

**Table 4:** mAP, average mAP, SD, and *η* value achieved by the Resnet50 backbone network of the SSD in detecting different blood cells in smear images when the combinations of algorithm hyperparameters in Table 3 were employed in three independent experimental runs

Experiment no	Dataset	mAP-Experiment no			Average mAP	SD	*η* value
		mAP-1	mAP-2	mAP-3			
1	Training set	0.7322	0.7194	0.7111	0.7209	0.0106	11.0848
	Test set	0.7179	0.7113	0.6713	0.7002	0.0252	10.4624
2	Training set	0.7715	0.772	0.7656	0.7697	0.0036	12.7541
	Test set	0.7705	0.7679	0.7672	0.7685	0.0017	12.7102
3	Training set	0.6245	0.6242	0.6247	0.6245	0.0003	8.5070
	Test set	0.6852	0.6853	0.6851	0.6852	0.0001	10.0393
4	Training set	0.1907	0.1902	0.1885	0.1898	0.0012	1.8282
	Test set	0.19	0.1896	0.1874	0.1890	0.0014	1.8196
5	Training set	0.0332	0.0332	0.0332	0.0332	0.0000	0.2933
	Test set	0.0373	0.0374	0.0372	0.0373	0.0001	0.3302
6	Training set	0.4613	0.4555	0.455	0.4573	0.0035	5.3083
	Test set	0.4729	0.472	0.4716	0.4722	0.0007	5.5501
7	Training set	0.6348	0.6332	0.6338	0.6339	0.0008	8.7288
	Test set	0.6961	0.695	0.6938	0.6950	0.0012	10.3131
8	Training set	0.7082	0.7161	0.733	0.7191	0.0127	11.0290
	Test set	0.6913	0.719	0.7311	0.7138	0.0204	10.8666
9	Training set	0.7767	0.7797	0.7821	0.7795	0.0027	13.1318
	Test set	0.7653	0.7646	0.7686	0.7662	0.0021	12.6219

### Inferring and conducting the best combination of factor levels of algorithm hyperparameters for the Resnet50-SSD model for exploring the detection accuracy

**Fig. 3:**
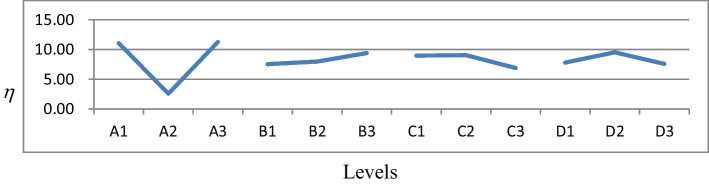
Plots of factor effects in the Resnet50 backbone network of the SSD

Table 5 shows the response table of each factor in the Resnet50 backbone network of the SSD, and Fig. 3 is the influence curve of each factor in the Resnet50 backbone network of the SSD, which were got by calculating the *η* value for each factor level in Table 4. Table 5 shows that factor levels 3, 3, 2, and 2 were selected for factors A, B, C, and D, respectively. Thus, the best combination of factor levels was A3: adam, B3: 18, C2: 10^−4^, and D2: 40 for the Resnet50 backbone network of the SSD.

**Table 5:** Response table of each factor in the Resnet50 backbone network of the SSD

Level	Factors			
	A	B	C	D
1	11.0706	7.5317	8.9597	7.8048
2	2.5666	7.9690	9.0506	9.5244
3	11.2672	9.4037	6.8942	7.5752
Effect	8.7006	1.8721	2.1564	1.9493
Maximum	11.2672	9.4037	9.0506	9.5244
Best level number	3	3	2	2
Best level value	adam	18	10^−4^	40

**Fig. 4:**
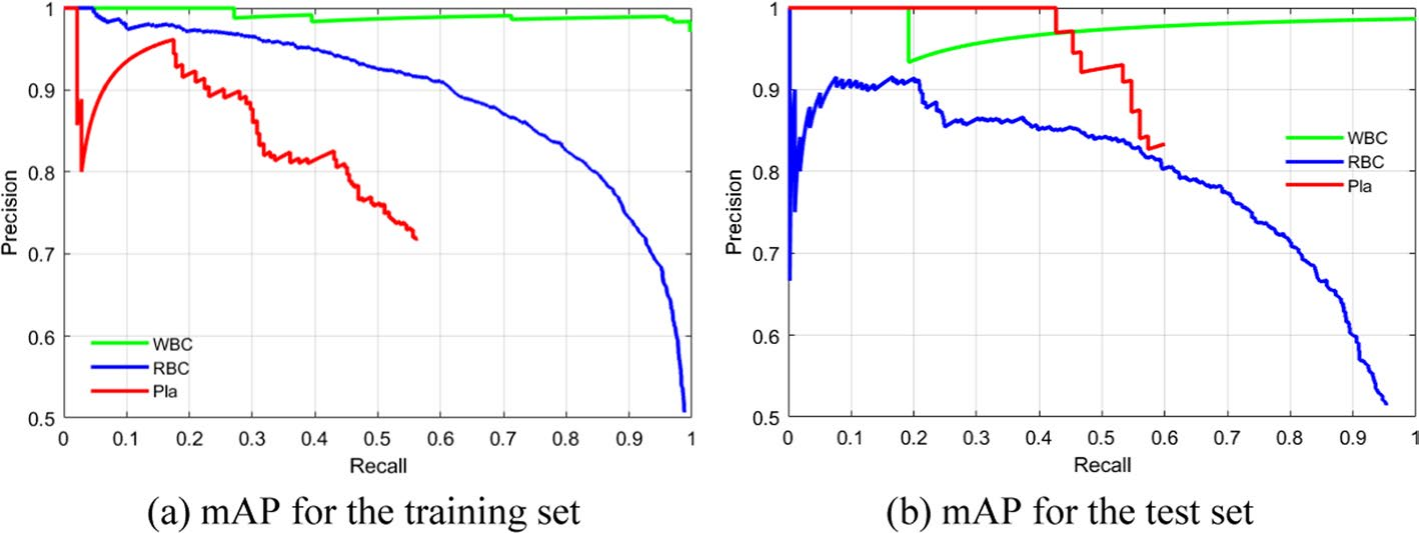
The mAP-1 case in Table 6 for the training and test sets in detecting different blood cells in microscopic smear images by using the Resnet50-SSD model

In the validation experiment, the best algorithm hyperparameters combination (i.e., A3: adam, B3: 18, C2: 10^−4^, and D2: 40) was employed in three independent experimental runs of the Resnet50-SSD model for detecting different blood cells in microscopic smear images. Table 6 shows the mAP, average mAP, SD, and *η* value achieved by the Resnet50-SSD model in the three independent experimental runs for the training and test sets in detecting different blood cells in microscopic smear images. The average mAP and *η* value obtained by the Resnet50-SSD model performed on the test set were 0.7747 and 12.9461, respectively, which exceeded those in each *L*_9_(3^4^) OA experiment (Table 4) on the test set. Figure 4 shows the mAP-1 case in Table 6 for the training and test sets in detecting different blood cells in microscopic smear images by using the Resnet50-SSD model. The mAP-1 for the test set in Table 6 and Fig. 4b was 0.7763, and the APs for the WBCs, RBCs, and platelets were 0.9766, 0.7627, and 0.5895, respectively. This detection accuracy was more accurate for the WBC class, because WBCs have clearly characterized and the largest size (platelet: RBC: WBC = 0.2:1:2 in diameter ratio) of different blood cells in the BCCD dataset. The detection accuracy of RBCs and platelets is relatively poor, because RBCs are most many but overlapping, and platelets are the smallest and overlap with RBCs without borders.

**Table 6:** mAP, average mAP, SD, and *η* value achieved by the Resnet50-SSD model in detecting different blood cells in smear images when the best algorithm hyperparameters combination was used in three independent experimental runs

Model	Dataset	mAP-Experiment no			Average mAP	SD	*η* value
		mAP-1	mAP-2	mAP-3			
Resnet50-SSD	Training set	0.7899	0.7866	0.7808	0.7858	0.0046	13.3823
	Test set	0.7763	0.7772	0.7707	0.7747	0.0035	12.9461

The best algorithm hyperparameters combination from the response table (Table 5) yielded the best result, even if not all combinations of factor levels were tested. Therefore, the best algorithm hyperparameters combination got from the validation experiments was employed for the Resnet50-SSD model to detect different blood cells in microscopic smear images.

### Analyzing the influence algorithm hyperparameters of the Resnet50-SSD model for exploring the detection accuracy in microscopic smear images

ANOVA was performed to understand what factors had the greatest influence on the accuracy of the Resnet50-SSD model in detecting different blood cells in microscopic smear images. Table 7 shows the ANOVA results. Factors A (Optimizer) had the greatest effect on detection accuracy. The percentage contribution of factor A to experimental variance was 87.33%. The second effect on accuracy was factor C (InitialLearnRate), which had 5.26% percentage contribution. Factors B (3.39%) and D (4.02%) had smaller percentage contributions. That is, in the selected levels for the four factors, a statistically significant factor in the accuracy of the Resnet50-SSD model in detecting different blood cells in microscopic smear images was ‘adam’.

**Table 7:** The ANOVA results

Factor	Sum of squares	Degrees of freedom	Variance	Expected sum of squares	Percentage contribution (%)
A: Optimizer	148.0572	2	74.0286	148.0572	87.33
B: MiniBatchSize	5.7544	2	2.8772	5.7544	3.39
C: IinitialLearnRate	8.9246	2	4.4623	8.9246	5.26
D: LearnRateDropPeriod	6.8096	2	3.4048	6.8096	4.02
Error	0.0000	0	0	0	
*S*_*T*_	169.5457	8		169.5457	100

## Discussion

Does the image size affect the accuracy of object detection in detecting different blood cells in microscopic smear images? The diameter ratio of platelets, RBCs, and WBCs is 0.2:1:2. Figure 1 shows the size and number of blood cells. RBCs are most many but overlapping, platelets are few but the smallest, and WBCs are the least but the largest. To understand the detection accuracy of the Resnet50-SSD model, each image was processed as 512 × 512 × 3 and 300 × 300 × 3 image sizes. Table 8 shows the comparing results of different image sizes by using the Resnet50-SSD model. The detection accuracy (0.7747 average mAP on the test set) of large images (512 × 512 × 3 image size) is higher than (0.7094 average mAP on the test set) that of smaller images (300 × 300 × 3 image size). For 512 × 512 × 3 image size, for example, the mAP-1 for the test set in Table 8 is 0.7763, and the APs for the WBCs, RBCs, and platelets are 0.9766, 0.7627, and 0.5895, respectively. For 300 × 300 × 3 image size, for example, the mAP-3 for the test set in Table 8 is 0.7102, and the APs for the WBCs, RBCs, and platelets are 0.9733, 0.7679, and 0.3893, respectively. As the detection image becomes smaller, the detection accuracy of small objects (e.g. platelets) is reduced. In the example, when the image size is 512 × 512 × 3, the detection accuracy for platelets is 0.5895, but when the image size is 300 × 300 × 3, the detection accuracy for platelets drops to 0.3893. Therefore, when there are differences in the size of the detected objects, the larger the image, the better the detection accuracy, under the permission of computing resources.

**Table 8:** Comparing results of different image sizes by using the Resnet50-SSD model

Image size	Dataset	mAP-Experiment no			Average mAP	SD	*η* value
		mAP-1	mAP-2	mAP-3			
512 × 512 × 3	Training set	0.7899	0.7866	0.7808	0.7858	0.0046	13.3823
	Test set	0.7763	0.7772	0.7707	0.7747	0.0035	12.9461
300 × 300 × 3	Training set	0.741	0.7389	0.7475	0.7425	0.0045	11.7833
	Test set	0.7081	0.7099	0.7102	0.7094	0.0011	10.7341

Is choosing a combination of hyperparameters necessary for object detection? This study found that an appropriate algorithm hyperparameters combination for the Resnet50-SSD model is essential for accurately detecting different blood cells in microscopic smear images. Table 4 shows average mAPs of the experiments 4, 5, and 6 for the test set are lower than 0.5 due to the poor combinations of algorithm hyperparameters for the Resnet50-SSD model. Additionally, the hyperparameters combination (e.g., Optimizer of ‘sgdm’, MiniBatchSize of 16, InitialLearnRate of 10^−1^, and LearnRateDropPeriod of 30), given by the Matlab example, was used in detecting different blood cells in microscopic smear images. Table 9 shows mAP, average mAP, SD, and *η* value achieved by the Resnet50-SSD model by using 512 × 512 × 3 image size in detecting different blood cells in microscopic smear images when the algorithm hyperparameters combination given by the Matlab example was used in three independent experimental runs. The average mAP of 0.7475 in Table 9 was still less than that of 0.7747 in Table 8, got by the proposed Resnet50-SSD model on the test set in this study. The results of this study indicate that a poor algorithm hyperparameters combination for the Resnet50-SSD model cannot accurately detect blood cells in microscopic smear images. Therefore, the study used a systematic Taguchi method to find the better algorithm hyperparameters combination for the Resnet50-SSD model for blood cells detection.

**Table 9:** Results achieved by the Resnet50-SSD model by using 512 × 512 × 3 image size in detecting different blood cells in microscopic smear images when the hyperparameters combination given by the Matlab example was used in three independent experimental runs

Model	Dataset	mAP-Experiment no			Average mAP	SD	*η* value
		mAP-1	mAP-2	mAP-3			
Resnet50-SSD	Training set	0.7374	0.7408	0.7326	0.7369	0.0041	11.5987
	Test set	0.7383	0.757	0.7471	0.7475	0.0094	11.9536

Alam and Islam [5] reported that the proposed Tiny YOLO, VGG-16 with YOLO, ResNet50 with YOLO, InceptionV3 with YOLO, and MobileNet with YOLO models achieved the mAPs of 0.6236, 0.7132, 0.7437, 0.6826, and 0.5207, respectively, on the test set. Although Alam and Islam [5] used the same BCCD database for research, they changed the data, used different image sizes, and had other numbers of training and test sets. Those mAPs got by the proposed models of Alam and Islam [5] were still lower than the average mAP of 0.7747 got by the proposed Resnet50-SSD model in this study, but comparing detection accuracy with each other is not reasonable, because both had different conditions. In addition, various researchers used their own datasets to detect and count blood cells, so comparisons are also not possible. Furthermore, all relevant studies reported to date have used small datasets. The use of large datasets is critical for accurate evaluation of state-of-the-art detection techniques.

**Fig. 5:**
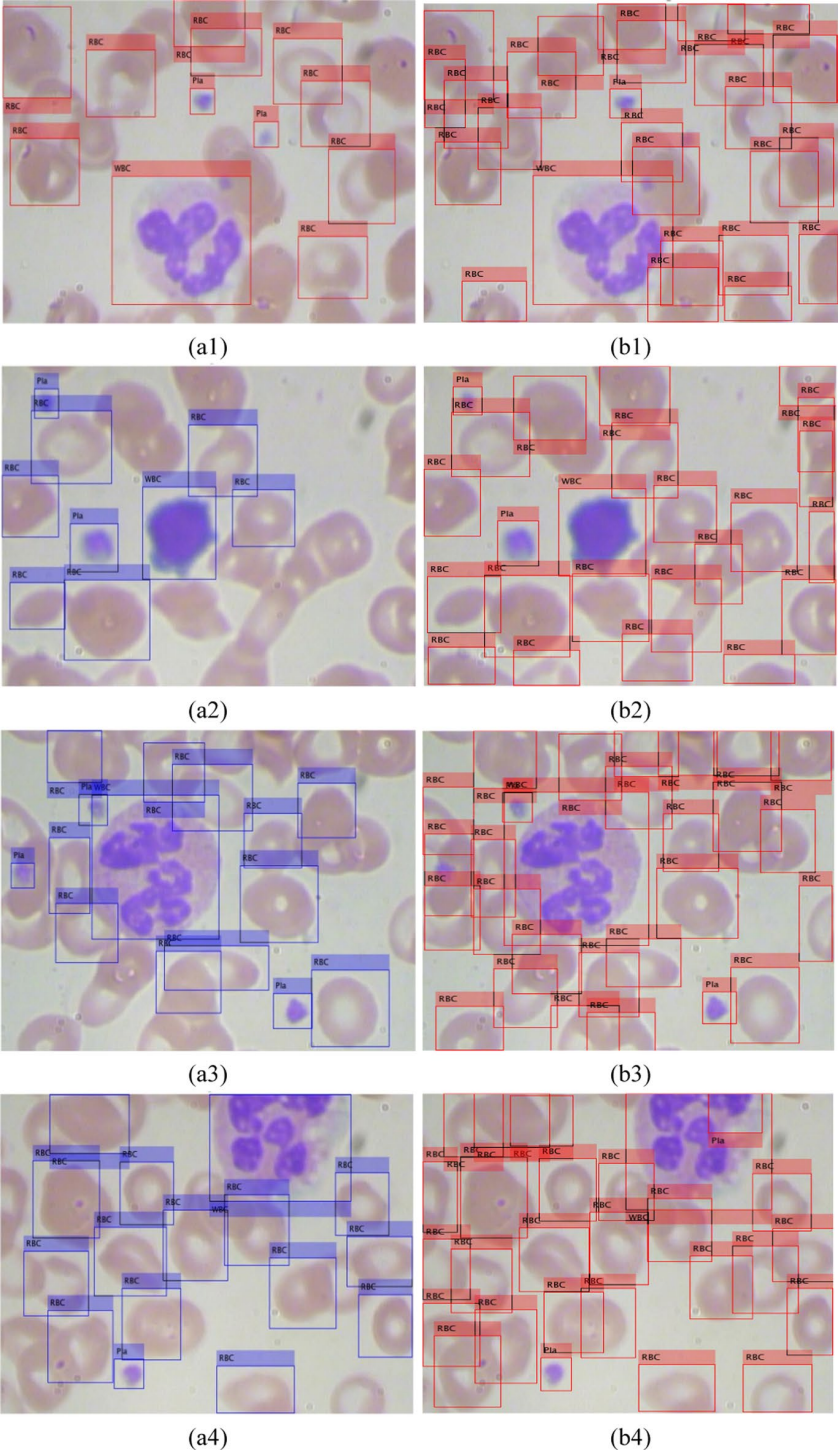
Ground truth labels of blood cells (**a1**–**a4**) and the labeled blood cells (**b1**–**b4**) detected by the proposed Resnet50-SSD model

For 512 × 512 × 3 image size, the mAP-1 for the test set in Table 8 is 0.7763. On the test set, there are 73 images and 994 ground truth labels across 3 classes, including 73 WBCs, 846 RBCs, and 75 platelets labels. The proposed Resnet50-SSD model can detect and count 75 WBCs (over 100% accuracy) in microscopic smear images, because WBCs are the largest and clearly characterized, and the number of ground truths of WBCs is less than the number of actual WBCs. In the detection and counting of RBCs using the proposed Resnet50-SSD model, the number of detected RBCs of 1628 is greater than the number of ground truths of RBCs of 846. The accuracy of counting RBCs is over 100%, because the number of ground truths of RBCs is less than the number of actual RBCs. The proposed Resnet50-SSD model can detect and count 57 platelets (76% accuracy) in microscopic smear images of the test set, because some platelets overlap with RBCs without borders. For example, Fig. 5 shows ground truth labels of blood cells and the labeled blood cells detected by the Resnet50-SSD model. The accuracy of detection and counting increases if the selected area has a better distribution of blood cells in microscopic smear images.

## Conclusions

The Resnet50-SSD model proposed in this study had better accuracy in identifying and counting blood cells (especially WBCs and RBCs) in microscopic smear images of blood cells. The first contribution of this study is to confirm that the image size affects the object detection accuracy in detecting different blood cells in microscopic smear images. The larger the image, the better the detection accuracy, when there are differences in the size of the detected objects. The detection accuracy of the Resnet50-SSD model with 512 × 512 input images in three independent experiments achieved 0.7747 average mAP was better than that of the Resnet50-SSD model with 300 × 300 input images achieved 0.7094 average mAP on the test set of blood cells images. The second contribution of this study is to confirm that an appropriate algorithm hyperparameters combination for the Resnet50-SSD model can obtain high image detection accuracy. The detection accuracy of the Resnet50-SSD model using the algorithm hyperparameters combination obtained by the Taguchi method achieved 0.7747 average mAP was better than that of the Resnet50-SSD model using the algorithm hyperparameters combination given by the Matlab example achieved 0.7475 average mAP on the test set of blood cells images. The third contribution of this study is to confirm that a systematic Taguchi experimental method can find the better algorithm hyperparameters combination for a backbone network of the SSD. In the validation experiment, the best algorithm hyperparameters combination (i.e., A3: adam, B3: 18, C2: 10^−4^, and D2: 40) was employed in three independent experimental runs of the Resnet50-SSD model for detecting different blood cells in microscopic smear images. The average mAP and *η* value obtained by the Resnet50-SSD model performed on the test set were 0.7747 and 12.9461, respectively, which exceeded those in each *L*_9_(3^4^) OA experiment (Table 4) on the test set. The fourth contribution of this study is to confirm that a better object detection model can find new objects that exceed the ground truth labels on the test set. The proposed Resnet50-SSD model can detect and count both WBCs and RBCs over 100% accuracy, because the number of ground truths of both WBCs and RBCs is less than the number of actual WBCs and RBCs on the test set.

## Methods

The research procedures included collecting and processing smear images of different blood cells for object detection, selecting the Resnet50 backbone network and algorithm hyperparameters for the SSD, designing combinations of algorithm hyperparameters of the Resnet50 backbone network for object detection using Taguchi method, conducting detecting experiments on smear images of different blood cells, recording blood cells detection performances of the Resnet50-SSD model, inferring the best combination of factor levels of algorithm hyperparameters for the Resnet50-SSD model, conducting the best algorithm hyperparameters combination of the Resnet50-SSD model for exploring the detection accuracy and counting different blood cells, and, finally, analyzing the algorithm hyperparameters of the Resnet50-SSD model for exploring the detection accuracy in microscopic smear images. The detailed steps are as follows.

### Collecting and processing smear images of different blood cells for object detection

The microscopic smear images got by the BCCD dataset have 364 annotated images, in which the width and height of the JPEG smear image are 640 and 480 pixels, respectively [20]. Each experiment randomly selected 291 images (80% of all images) as the training set and 73 images (the remaining 20% images) as the test set for identifying and counting different blood cells. The ground truth labels for the training and test sets were generated for evaluating the detection accuracy of supervised learning techniques. To efficiently detect objects and fit into the backbone network of SSD, each image was processed as 512 × 512 × 3 image size, where 3 is the number of color channels.

Data augmentation was used to randomly transform the original data during the training process to improve model accuracy. The diversity of training data can be added by using data augmentation without actually having to increase the number of labeled training samples. Data augmentation strategies included randomly scaling the image and associated box labels, randomly flipping the image and associated box labels horizontally, and using jitter image colors. The test data did not use data augmentation. Ideally, the test data should represent the original data and be unmodified for unbiased evaluation.

### Selecting the Resnet50 backbone network and algorithm hyperparameters for the SSD

**Fig. 6:**
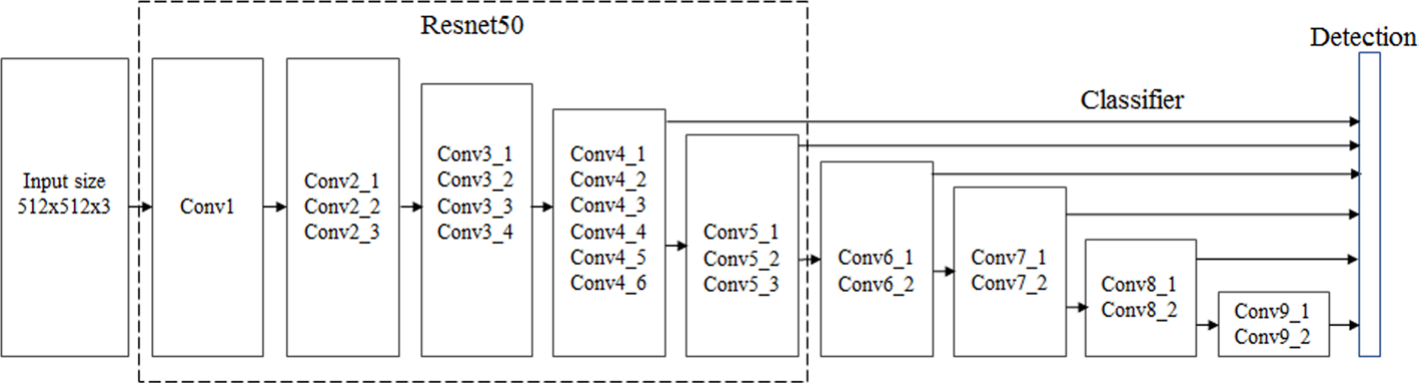
The Resnet50 network as a backbone network for the SSD

The Resnet, proposed by He et al. [21], was the winner in the 2015 ImageNet large-scale visual recognition challenge (ILSVRC) [22] in image classification (achieved a Top-5 error rate of 3.57%), detection, and localization, as well as winner of MS COCO 2015 segmentation and detection. Therefore, the Resnet50 was selected as a backbone network of the SSD for the study for detecting and counting different blood cells, because the Resnet had excellent feature extraction ability. Figure 6 shows the Resnet50 network as a backbone network for the SSD.

In order for the Resnet50 network to achieve high accuracy in detecting images, it is crucial to choose the proper combination of algorithm hyperparameters that are set before the training process starts. In this study, the algorithm hyperparameters for the Resnet50 backbone network of the SSD were Optimizer, MiniBatchSize, InitialLearnRate, and LearnRateDropPeriod. Optimizer was the training option. MiniBatchSize was a mini-batch at each iteration. InitialLearnRate was an initial learning rate during training. LearnRateDropPeriod was the number of the learning rate drop period. Additionally, LearnRateDropFactor, a factor for dropping the learning rate, was set 0.8, MaxEpochs, the maximum number of training epochs, was set 200, and LearnRateSchedule, an option for dropping the learning rate during training, was to be 'piecewise'. The learning rate (LearnRate) was equal to the learning rate in the previous period multiplied by the number of the learning rate drop period (i.e., LearnRateDropPeriod).

### Designing combinations of algorithm hyperparameters of the Resnet50 backbone network for object detection using Taguchi method

The Taguchi method is a statistical experimental method for evaluating and implementing process and product improvements. The rationale for this approach is to improve quality by minimizing the causes of variation instead of eliminating them. The Taguchi method minimizes the number of experiments required to study many design variables. An efficient way to study the effects of multiple control factors simultaneously is to arrange the matrix experiments in an orthogonal array (OA). A better combination of factor levels is found by OA and signal-to-noise ratio (SNR) [23–26].

In this study, the four algorithm hyperparameters for the Resnet50 backbone network were Optimizer, MiniBatchSize, InitialLearnRate, and LearnRateDropPeriod. To account for nonlinear effects and minimize the number of experiments required, a three-level *L*_9_(3^4^) OA was used. Therefore, the algorithm hyperparameters combinations got by the three-level *L*_9_(3^4^) OA were used in the Resnet50 backbone network for detecting and counting different blood cells in microscopic smear images.

### Conducting detecting experiments on smear images of different blood cells and recording blood cells detection performances of the Resnet50-SSD model

The results of blood cells detection recorded for the training and test sets included 1) mean average precision (mAP) of each experimental run, 2) average mAP in three independent runs, 3) the mAP standard deviation of the three independent runs and 4) *η* value.

AP is defined as finding the area under the precision-recall curve for one class. When performing information retrieval tasks, precision is a measure of the relevance of the results. Precision is computed as the positive predictive value (true positives divided by true positives plus false positives). Another measure of information retrieval performance is recall (sensitivity), which is computed as true positive rate (true positives divided by true positives plus false negatives). The value of mAP comes from calculating the AP for each class. The average AP for all classes is mAP. The concept of SNR was first applied to communications and then to engineering. For engineering applications, a larger SNR (*η*) is preferable and shows better performance. Taguchi recommended multiplying the common logarithm of SNR by 10 to get the SNR in decibels (dB). In this study, the equation of the smaller-the-better characteristic is $\eta \; = \; - 10\;\log \;(\overline{y} - m)^{2}$, where $\overline{y} \; = \;\;\frac{1}{n}\sum\limits_{t = 1}^{n} {y_{t}^{{}} }$ (a set of data *y*_1_, *y*_2_, …, *y*_n_, *y*_*t*_ represents the mAP of model training and prediction in each experiment) and *m* = 1 (i.e., the mAP of the target is 100%).

### Inferring the best combination of factor levels of algorithm hyperparameters for the Resnet50-SSD model

A response table was used to find the best combination of factor levels for the algorithm hyperparameters by using the three-level *L*_9_(3^4^) OA and *η* values. To construct the response table, the effects of different factors were as follows: *E*_*fl*_ = the mean of the sum of *η*_*i*_ for factor *f* at level *l*, where *f* is the factor name, *l* is the level number, and *i* is the experiment number. The response table was used to investigate the *η* for each factor level after 9 experiments of the three-level *L*_9_(3^4^) OA. The response table displayed the mean *η* for each factor level and the maximum mean *η* for each factor. The main purpose was to use the response table to find the best level for each factor. The best level was defined as the level with the highest *E*_*fl*_ value in the experimental area. That is, the best combination of factor levels for the algorithm hyperparameters was inferred from the results of 9 experiments, although not all combinations of factor levels for the algorithm hyperparameters were considered (i.e., 3^4^ experiments).

### Conducting the best algorithm hyperparameters combination of the Resnet50-SSD model for exploring the detection accuracy and counting different blood cells

The best combination of factor levels of algorithm hyperparameters got by the response table for the Resnet50-SSD model was employed to detect different blood cells.

### Analyzing the influence algorithm hyperparameters of the Resnet50-SSD model for exploring the detection accuracy in microscopic smear images of blood cells

The Taguchi experimental process used analysis of variance (ANOVA) to find significant controlling factors by performing a minimal number of experiments. ANOVA was employed to find the algorithm hyperparameter that significantly affected the most important characteristic in the Resnet50-SSD model, namely the detection accuracy of different blood cells in microscopic smear images.

## Data Availability

All data obtained and analyzed in this study are included in this article. Microscopic smear images of blood cells were got from the BCCD dataset available online at https://github.com/Shenggan/BCCD_Dataset

## Abbreviations

BCCD: Blood cell count and detection
SSD: Single shot detector
WBCs: White blood cells
RBCs: Red blood cells
mAP: Mean average precision
ANOVA: Analysis of variance
